# Bone Maturation as a Predictive Factor of Catch-Up Growth During the First Year of Life in Born Small for Gestational Age Infants: A Prospective Study

**DOI:** 10.3389/fendo.2020.00147

**Published:** 2020-03-24

**Authors:** Giorgia Pepe, Mariarosa Calafiore, Mariella Valenzise, Domenico Corica, Letteria Morabito, Giovanni Battista Pajno, Tommaso Aversa, Malgorzata Wasniewska

**Affiliations:** Unit of Pediatrics, Department of Human Pathology of Adulthood and Childhood, University of Messina, Messina, Italy

**Keywords:** small for gestational age, preterm infant, bone maturation, distal femoral epiphyseal nucleus, catch-up growth

## Abstract

**Background:** About 85–90% of children born small for gestational age (SGA) experience a catch-up growth that occurs mostly during the first year of life and results in a full stature recovery by the age of 2.

**Objective:** To investigate the relation between bone maturation (BM) and catch-up growth during the first year of life in SGA infants.

**Method:** Newborns whose weight and/or length was <-2 SD for gestational age were classified as SGA (group A). The study included a group of 32 SGA, 21 of which are full term [37–41 gestation weeks (GW), subgroup A1] and 11 preterm (30–36 GW, subgroup A2). Control group (B) consisted of 19 full-term and adequate-for-gestational-age (AGA) newborns. All the participants were born in the same hospital and period (January–December 2017). Chromosomal disorders, congenital defects, and maternal chronic diseases were criteria of exclusion. The study population underwent longitudinal evaluation of growth parameters and BM at 0, 3, 6, and 12 months. Assessment of BM was performed by an ultrasonographic (US) study of Béclard's nucleus (NB) (<3 mm at birth, meaning intrauterine delay of BM).

**Results:** Significantly higher height velocity (HV) was observed in subgroup A2 vs. A1 (32.4 ± 8.0 vs. 25.6 ± 2.9 cm, *p* = 0.01); nevertheless, more subjects in subgroup A2 had height <-2 SD at year 1 than had subgroup A1 (27.3 vs. 0%, *p* = 0.01). Intrauterine delay of BM was more common in group A vs. B (59.4 vs. 21.2%, *p* = 0.0078) and in subgroup A2 vs. A1 (90.9 vs. 42.9%, *p* = 0.0086). In group A, HV over the first year of life negatively correlates with NB diameter assessed at birth (*r* = −0.6, *p* < 0.001) but positively correlates with NB growth (*r* = 0.52, *p* < 0.01). Moreover, SGA babies with intrauterine delay of BM showed higher HV and better height gain at 12 months' evaluation than did SGA with adequate BM (29.75 ± 3.1 vs. 23.8 ± 2.7 cm, *p* = 0.003).

**Conclusion:** Neonatal BM should be regarded as a predictive factor of SGA height gain during the first year of life. US evaluation of NB is a useful noninvasive technique to identify intrauterine delay of BM, which positively correlates with early postnatal catch-up growth of SGA infants.

## Introduction

The term small for gestational age (SGA) describes neonates whose weight and/or length at birth is below the cutoff value of −2 SD for gestational age. In clinical practice, the definition of SGA requires rigorous evaluations: (a) gestational dating, assessed by first-trimester ultrasound exam; (b) accurate anthropometry at birth, including measurements of weight, length, and head circumference; and (c) comparison with data coming from adequate reference population (1–3).

Instead, the concept of intrauterine growth restriction (IUGR) refers to slow fetal growth due to maternal, fetal, and/or placental causes, on the basis of at least two ultrasound exams performed during pregnancy. This process may result in a SGA newborn, even if it is worth noting that a SGA baby is not necessarily IUGR. Indeed, the SGA definition can also include a percentage of neonates (18–22%) who are only constitutionally small (4).

The recognition of SGA condition raises public health issues, as it could imply a variety of short- and long-term consequences including increased perinatal morbidity and mortality, higher risk of short stature, metabolic disorders later in life, and neurocognitive vulnerabilities (2, 5, 6).

With respect to growth, it is well documented that about 85–90% of SGA babies experience a catch-up growth that occurs mostly during the first 12 months of life and results in a full stature recovery by the age of 2 years. The remaining 10–15% of SGA children do not undergo compensatory growth and will remain permanently <-2 SD for height. Indeed, 20% of short-stature adults were born SGA. Finally, the preterm infants born SGA are regarded as a special case, as they may take up to 4 years to achieve a height within the normal range for population (1, 7, 8). The mechanisms of this accelerated linear growth are still not completely clarified (9). Birth weight, birth length, the magnitude of fetal growth retardation and midparental height could be regarded as influencing factors (8, 10, 11).

To the best of our knowledge, bone maturation (BM) assessment in SGA newborns has never been reported to now. As a consequence, there is a lack of specific literature data about the potential influence of BM progression on compensatory growth in this population.

Our prospective study aims to detect the factors that may be involved in early catch-up growth and to investigate the link between BM and postnatal growth during the first year of life in SGA infants.

## Materials and Methods

### Study Population

The study population consisted of 32 SGA neonates who were born in the same hospital and period (January–December 2017), fulfilling the following inclusion criteria: (a) birth weight and/or length <-2 SD for gestational age; and (b) gestational dating assessed by first-trimester ultrasound exam.

Sick newborns and those with genetic syndromes, chromosomal disorders, congenital defects, neonatal screening positivity, or maternal chronic diseases were excluded from the study.

The entire series of SGA newborns (group A, 18 males) was divided into two subgroups, according to gestational age: 21 full-term SGA [37–41 gestation weeks (GW)] were included in subgroup A1 (11 males) and 11 SGA preterm (30–36 GW) in subgroup A2 (7 males). According to BM assessment at birth, group A was divided into two further subgroups: 13 SGA with adequate BM were part of subgroup A3 and 19 SGA with delayed BM of subgroup A4. The SGA population was compared with controls (group B, 13 males) represented by 19 full-term and adequate-for-gestational-age (AGA) neonates born in the same hospital and period.

### Study Design

Newborns who fulfilled the above reported inclusion criteria were enrolled for this prospective one-center study.

The study population underwent longitudinal evaluation of growth parameters (weight, length, and head circumference) and BM at birth and at 3, 6, and 12 months. Bone age was assessed at 12 months of age.

The study design was approved by the local ethical committee of the hospital participating to the study. Informed consent was obtained earlier by the children's parents.

### Methods

Gestational age was defined by ultrasound measurement recorded during early pregnancy. Newborns whose weight and/or length was <-2 SD for gestational age were classified as SGA. Anthropometric evaluation was based on standard length, weight, and head circumference measurements. To allow the comparison between different ages and genders, the above-mentioned auxological parameters were expressed as standard deviation scores (SDSs). As recommended by the Italian Society of Pediatric Endocrinology and Diabetology (ISPED), the reference population standards were assessed according to the INeS (Italian Neonatal Study) charts from birth to term (12, 13) and to World Health Organization (WHO) growth charts from term to 24 months (14). Assessment of BM was performed by an ultrasonographic (US) study of the distal femoral epiphyseal ossification center known as Béclard's nucleus (NB) (15, 16). This approach had been previously used for assessment of congenital hypothyroidism, where BM retardation at birth turned out to be a helpful indicator of intrauterine and severe hypothyroidism, with a negative prognostic value on a long-term intellectual outcome (17–21). In all the patients, the presence or absence of the distal femoral epiphyseal nucleus was scored according to the method proposed by Ilicki et al. (17), applied to US evaluation and simplified as follows: bony nucleus diameter was measured using electronic calipers, and BM at birth was considered adequate if NB diameter exceeded 3 mm or delayed if this bony nucleus was absent or its diameter was <3 mm (meaning intrauterine delay of BM). This definition was chosen according to the criteria suggested by Senecal et al. (18) and partially modified by Ilicki et al. (17). The same limit had previously been adopted by Heyerdahl et al. (19). In fact, a knee epiphysis diameter <3 mm implies that the epiphyseal area is <0.07 mm^2^, which is very similar to the cutoff value (epiphyseal area <0.05 mm^2^) used by Glorieux et al. (20). This method of BM evaluation had also been used by Wasniewska et al. (21). An echographic study of the distal femoral epiphyseal nucleus was performed with a commercial US machine (Esaote Healthcare Technologies) equipped with a high-frequency linear transducer (7.5–20 MHz). Bone age was estimated by left hand and wrist X-ray at the age of 1 year using Greulich and Pyle's comparative method (22).

### Statistical Analysis

Numerical data were expressed as mean, SD, and range; categorical variables were expressed as absolute frequency and percentage. For comparisons of two means, Student's *t*-test (normally distributed data) and the Mann–Whitney *U*-test (non-parametric data) were used. Frequency rates were compared by a chi-squared test. Correlations between quantitative variables were assessed using Pearson's correlation analysis. Statistical analyses were performed using IBM SPSS Statistics for Windows, Version 21 (Armonk, NY, IBM Corp.). A *p*-value smaller than 0.05 was considered statistically significant.

## Results

### Main Auxological and Clinical Data at Birth

The clinical features of the entire study population at birth are shown in Table 1. Mean Apgar score at 5 min, body length, and weight were significantly lower in group A than in group B. NB mean diameter at birth was also significantly lower in group A than in control group. Intrauterine delay of BM was documented more frequently in group A than group B (59.4 vs. 21.1%, *p* = 0.0078) and in subgroup A2 than A1 (90.9 vs. 42.9%, *p* = 0.0086).

**Table 1:** Mean ± SDSs and number (*n*) with percentage (%) data collected at birth in the overall study population.

	**Sex (*n*)**	**Apgar 5 min (mean ± SD)**	**Weight (SD)**	**Height (SD)**	**Delayed bone maturation *n* (%)**	**Béclard's nucleus (mm)**
Group A (*n* = 32)	M 18/32	9.1 ± 0.8	−2.1 ± 0.3	−1.8 ± 1.2	19 (59.4%)	2 ± 1.7
Group B (*n* = 19)	M 13/19	9.6 ± 0.5	−0.3 ± 1.1	0.2 ± 0.7	4 (21.1%)	3.3 ± 1.6
*p*	0.39	0.005	<0.0005	<0.0005	0.008	0.0095
Subgroup A1 (*n* = 21)	M 11/21	9.1 ± 0.7	−2 ± 0.7	−1.33 ± 0.88	9 (42.9%)	2.6 ± 1.6
Subgroup A2 (*n* = 11)	M 7/11	9 ± 0.9	−2 ± 0.75	−2.6 ± 1.2	10 (91%)	0.9 ± 1.3
*p* A1 vs. A2	0.75	0.74	0.26	0.002	0.0086	0.0059
*p* A1 vs. B	0.50	0.009	0.00069	0.00008	0.14	0.245

Comparison between groups A (SGA neonates) and B (AGA controls), subgroups A1 (SGA full term) and A2 (SGA preterm), and A1 vs. group B.

*SDSs, standard deviation scores; SGA, small for gestational age*.

### Growth Monitoring During the First Year of Life

**Figure 1:**
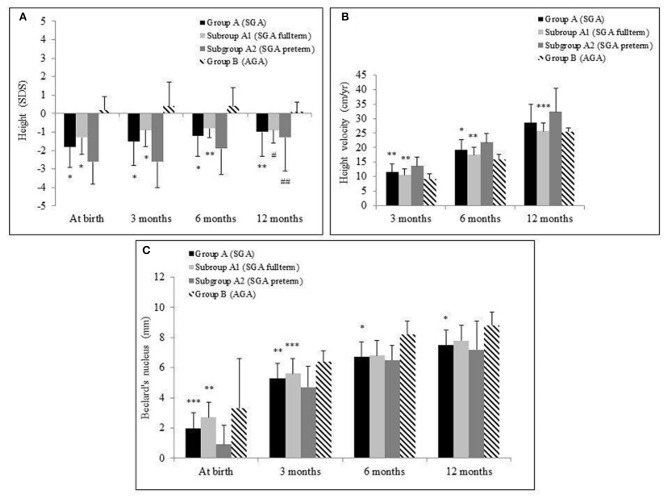
Mean and standard deviation of height standard deviation scores (SDSs) **(A)**, height velocity in cm/year **(B)**, and Béclard's nucleus in mm **(C)** in the groups A [small for gestational age (SGA)] and B [adequate for gestational age (AGA)] and in the subgroups A1 (SGA full term) and A2 (SGA preterm) during the 12-month follow-up. *p < 0.0005, **p < 0.005, ***p < 0.05. Comparison of group A vs. B and subgroup A1 vs. A2. ^#^*p* < 0.0005, ^##^*p* < 0.005. Comparison of 12-month value vs. at birth in each group.

Mean first-year height velocity (HV) of the entire population (group A + B) was 25.5 ± 13.2 cm. Significantly higher HV was observed in subgroup A2 vs. A1 (32.4 ± 8.0 vs. 25.6 ± 2.9 cm, *p* = 0.01); nevertheless, subjects in subgroup A2 presented more frequently with height <-2 SD than did subgroup A1 (27.3 vs. 0%, *p* = 0.01). HV was overall higher in group A, if compared with controls, but without reaching statistical significance [group A (28.6 ± 6.5 cm) vs. group B (25.5 ± 2.9 cm), *p* = 0.10]. The main features of growth pattern for each group and subgroup during follow-up are shown in Figures 1A,B. Subgroup A2 displayed overall a better catch-up growth pattern, which occurred later if compared with the earlier and more gradual stature recovery highlighted in subgroup A1. Indeed, subgroup A2 started catch-up growth only after 3 months of life, with significantly higher HV recorded after this period compared with baseline (*p* < 0.0005).

Group B exhibited a significantly higher body weight (SDSs) than group A during the entire follow-up period, without statistically significant difference between A1 and A2 subgroups.

US evaluation recorded a progressive NB growth during 12 months' follow-up in group A. Instead, group B showed this growth only until the ninth month of follow-up, with no further NB increase (Figure 1C). In group A, HV over the first year of life negatively correlated with NB diameter assessed at birth (*r* = −0.6, *p* < 0.001) but positively correlated with NB growth during follow-up (*r* = 0.52, *p* < 0.01). This relation was even stronger in subgroup A2 (respectively, *r* = −0.64, *p* < 0.01; *r* = 0.77, *p* < 0.01).

Growth parameters, BM, and bone age at the end of 12 months' follow-up are presented in Table 2.

**Table 2:** Mean ± SDSs of growth parameters collected at 12 months in the overall study population.

	**Weight (mean ± SD)**	**Height (mean ± SD)**	**Béclard's nucleus (mm)**	**Bone age (months)**
Group A	−1.6 ± 0.98	−1.04 ± 1.26	7.5 ± 1.07	8.9 ± 4.29
Group B	−0.45 ± 0.44	0.13 ± 0.92	8.8 ± 0.9	12.3 ± 4.57
*p*	0.0001	0.002	0.0001	0.0513
Subgroup A1	−1.52 ± 0.95	−0.85 ± 0.73	7.8 ± 1.0	9.7 ± 3.4
Subgroup A2	−1.67 ± 1.05	−1.28 ± 1.7	7.2 ± 1.9	7.9 ± 5.17
*p* A1 vs. A2	0.7213	0.4310	0.2443	0.3265
*p* A1 vs. B	0.0002	0.0034	0.0022	0.149

Comparison between group A (SGA neonates) and B (AGA controls), subgroups A1 (SGA full-term) and A2 (SGA preterm), and A1 vs. group B.

*SDSs, standard deviation scores; SGA, small for gestational age*.

### Growth Pattern in Small for Gestational Age Infants With Delayed Bone Maturation

**Figure 2:**
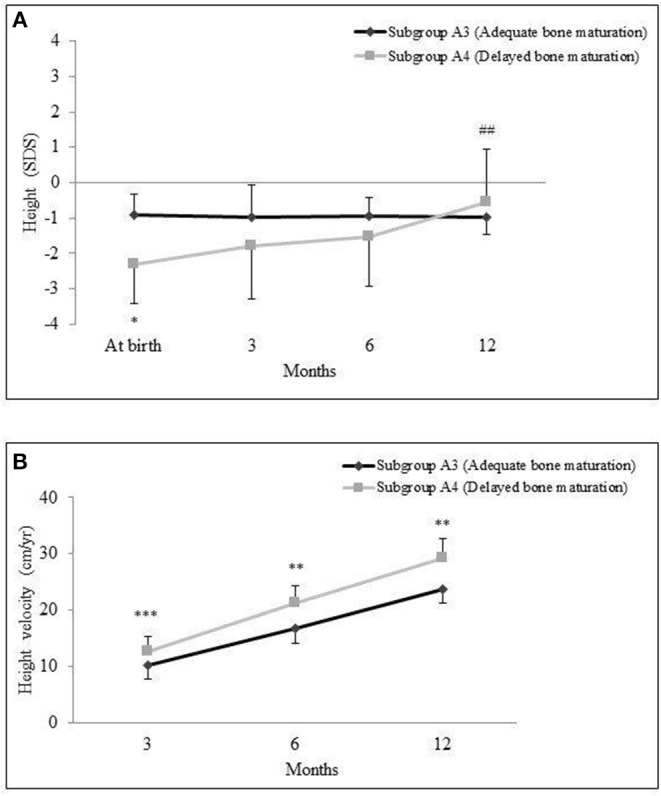
Mean and standard deviation of height standard deviation scores (SDSs) **(A)** and height velocity in cm/year and **(B)** in small for gestational age (SGA) babies classified into subgroups A3 (adequate bone maturation) and A4 (delayed bone maturation) during the 12-month follow-up. *p < 0.0005, **p < 0.005, ***p < 0.05. Comparison of subgroup A3 vs. A4. ^##^*p* < 0.0005. Comparison of 12-month values vs. at birth.

SGA infants with documented intrauterine delay of BM (subgroup A4) exhibited a pattern of progressive catch-up growth during the follow-up period. If compared with SGA infants with adequate BM (subgroup A3), subgroup A4 showed higher HV and better height gain (29.75 ± 3.1 cm in A4 subgroup vs. 23.8 ± 2.7 cm in A3 subgroup, *p* = 0.003) at 12 months' evaluation (Figures 2A,B).

Moreover, the frequency of a height outcome <-2 SD at the age of 1 year did not significantly differ in these two subgroups (15.8% in A4 subgroup vs. 0% in A3 subgroup; *p* = 0.13).

At the end of follow-up, bone age evaluation showed similar scores in both subgroups (8.1 ± 5.2 months in A4 subgroup vs. 8.4 ± 4.9 months in A3 subgroup, *p* = 0.92).

## Discussion

It is well known that SGA infants differ in postnatal growth. The typical growth pattern is characterized by a period of accelerated linear growth that occurs mainly in the first 12 months of life and results in a full stature recovery by the age of 2 years. However, 10–15% of SGA children do not undergo compensatory growth, achieving an adult height ~20 cm below their peers' and their target height (7, 8). The pathophysiology of postnatal growth failure is complex and involves a variety of alterations in the growth hormone (GH)/insulin-like growth factor (IGF)-1 axis. Classic GH deficiency is rarely described in SGA infants. In addition, mean IGF-1 and IGF binding protein-3 (IGFBP-3) levels can be reduced, underlying that the mechanisms responsible for growth failure may vary from insufficient IGF-1 production to IGF-1 insensitivity (6, 23). Nevertheless, there is no evidence that circulating levels of GH, IGF-1, and IGFBP-3 could be predictive of subsequent growth, and no biochemical markers have been identified yet (1, 9). During the first 6–12 months of life, spontaneous catch-up growth seems to be influenced predominantly by birth length, birth weight, gestational age, and the magnitude of growth retardation. Only later on (from 2 years of age onward) will the genetic influence of midparental height appear to take over (7, 24). Chromosomopathies or recognized syndromes (such as Silver–Russell or 3M) are associated with an incomplete catch-up growth (8, 10, 11, 25).

To our best knowledge, BM evaluation in SGA newborns has never been reported. It is therefore unknown if a delay of BM could be detected in SGA population at birth. Moreover, the possible influence of BM progression on SGA catch-up growth has never been studied until now.

Our prospective study aims to evaluate the auxological outcome during the first year of life in a cohort of SGA infants and to investigate the frequency of BM retardation at birth and whether BM delay may be involved in early catch-up growth. For this purpose, we chose a noninvasive technique of BM assessment. Indeed, ultrasonographic evaluation of the distal femoral epiphysis has been suggested as a method of determining skeletal maturity in newborn and infants, showing an excellent reliability and correlation with traditional radiological assessment (15, 16, 26).

SGA preterm infants showed overall a better catch-up growth pattern, which occurred later (after the third month of life) and with higher HV than observed in SGA full term, who exhibited instead an earlier and more gradual stature recovery.

In addition, our study showed that there is a positive relationship between BM at birth and HV during the first year of life in SGA infants. Indeed, intrauterine delay of BM positively correlates with catch-up growth, allowing higher HV and better height gain at 12 months' evaluation. These findings are even more evident in SGA preterm. Despite higher HV recorded among the above-mentioned subgroup, SGA preterm exhibited more frequently <-2 SD height outcome at the end of follow-up.

In conclusion, our results suggest for the first time that neonatal BM should be regarded as a predictive factor of SGA height gain during the first year of life. US evaluation of NB turned out to be a useful noninvasive technique to identify intrauterine delay of BM, which seems to positively correlate with early postnatal catch-up growth of SGA infants.

Limitations of the present study were represented by the small sample size; however, this weakness is counterbalanced by the high level of homogeneity exhibited by our population. In addition, our control group included only AGA full term, so it could be considered as only partially representative for the total study population (SGA full term and preterm).

Taking into consideration the lack of specific literature data on neonatal BM and its relationship with gestational age, we could not exclude that the delayed BM of SGA preterm infants in the present study could be related to prematurity. Overall, our data seem to encourage the use of US evaluation of NB, especially among neonates <-2 SD of length at birth. Anyway, to further characterize the influence of BM on SGA catch-up growth, future studies on a wider population are needed.

## Data Availability Statement

All datasets generated for this study are included in the article/supplementary material.

## Ethics Statement

The studies involving human participants were reviewed and approved by Ethics Committee of Messina. Written informed consent to participate in this study was provided by the participants' legal guardian/next of kin.

## Author Contributions

MW and MC conceived the manuscript. GP, MC, and DC were involved in data collection. GP, MW, and LM were involved in writing of the manuscript and carried out data analysis. MV, GBP, and TA were involved in literature search. All authors approved the submitted version of the manuscript.

### Conflict of Interest

The authors declare that the research was conducted in the absence of any commercial or financial relationships that could be construed as a potential conflict of interest.
